# Integration of *Salmonella* into Combination Cancer Therapy

**DOI:** 10.3390/cancers13133228

**Published:** 2021-06-28

**Authors:** Besan H. Al-Saafeen, Maria J. Fernandez-Cabezudo, Basel K. al-Ramadi

**Affiliations:** 1 Department of Medical Microbiology and Immunology, College of Medicine and Health Sciences, United Arab Emirates University, Al Ain 17666, United Arab Emirates; 201970008@uaeu.ac.ae; 2 Department of Biochemistry and Molecular Biology, College of Medicine and Health Sciences, United Arab Emirates University, Al Ain 17666, United Arab Emirates; mariac@uaeu.ac.ae; 3 Zayed Center for Health Sciences, United Arab Emirates University, Al Ain 17666, United Arab Emirates

**Keywords:** *Salmonella*, cancer, combination therapy, immunotherapy

## Abstract

**Simple Summary:**

Despite significant advances in the development of new treatments, cancer continues to be a major public health concern due to the high mortality associated with the disease. The introduction of immunotherapy as a new modality for cancer treatment has led to unprecedented clinical responses, even in terminal cancer patients. However, for reasons that remain largely unknown, the percentage of patients who respond to this treatment remains rather modest. In the present article, we highlight the potential of using attenuated *Salmonella* strains in cancer treatment, particularly as a means to enhance therapeutic efficacy of other cancer treatments, including immunotherapy, chemotherapy, and radiotherapy. The challenges associated with the clinical application of *Salmonella* in cancer therapy are discussed. An increased understanding of the potential of *Salmonella* bacteria in combination cancer therapy may usher in a major breakthrough in its clinical application, resulting in more favorable and durable outcomes.

**Abstract:**

Current modalities of cancer treatment have limitations related to poor target selectivity, resistance to treatment, and low response rates in patients. Accumulating evidence over the past few decades has demonstrated the capacity of several strains of bacteria to exert anti-tumor activities. *Salmonella* is the most extensively studied entity in bacterial-mediated cancer therapy, and has a good potential to induce direct tumor cell killing and manipulate the immune components of the tumor microenvironment in favor of tumor inhibition. In addition, *Salmonella* possesses some advantages over other approaches of cancer therapy, including high tumor specificity, deep tissue penetration, and engineering plasticity. These aspects underscore the potential of utilizing *Salmonella* in combination with other cancer therapeutics to improve treatment effectiveness. Herein, we describe the advantages that make *Salmonella* a good candidate for combination cancer therapy and summarize the findings of representative studies that aimed to investigate the therapeutic outcome of combination therapies involving *Salmonella*. We also highlight issues associated with their application in clinical use.

## 1. Introduction

It is a well-known fact that cancer is a major public health concern worldwide, with nearly 10 million deaths attributed to this chronic disease each year [1]. Although chemotherapy and radiotherapy remain the “gold standard” for cancer treatment, they have been associated with inherent limitations including (a) lack of tumor selectivity and inadequate tissue penetration, (b) high toxicity to normal tissues, (c) development of resistance to treatment, and (d) tumor recurrence. In the past few years there has been unprecedented and durable success with the use of immune checkpoint blockade or chimeric antigen receptor T cell therapies (CAR-T) in treating cancer [2]. However, the clinical application of such approaches has encountered several challenges in association with the low response rate among treated patients in addition to the development of immune-related adverse effects [3]. These obstacles reduce the capacity of cancer therapies to achieve the optimum therapeutic outcome and therefore underscore the increasing demand to develop better strategies for cancer treatment.

Bacterial-mediated cancer therapy (BMCT) was recognized in the 19th century when Vautier first noticed tumor regression in cancer patients with gas gangrene [4]. Subsequently, William Coley succeeded in utilizing heat-inactivated *Streptococcus pyogenes* and *Serratia marcescens* to treat inoperable tumors, providing BMCT as one of the first examples of cancer immunotherapy [5,6]. Despite the achieved success, BMCT gradually disappeared from clinical use due to criticisms of Coley’s toxin along with the rise of chemotherapy and radiotherapy as effective cancer treatments. The development of molecular biology in addition to the increased understanding of host–pathogen interactions and tumor biology reinspired the interest of researchers toward utilizing bacteria as an anti-cancer therapeutic agent. Outstanding and promising candidates for BMCT include *Bacillus* Calmette-Guerin, *Listeria monocytogenes*, *Salmonella enterica* serovar Typhimurium (hereafter *S. typhimurium*), *Clostridium novyi-NT*, and *Escherichia coli* [7,8,9,10,11]. Several lines of evidence have documented the anti-tumor efficacy of the facultative anaerobic *Salmonella* in addition to its oncolytic capacity and immunomodulatory effects [12,13,14,15,16,17,18]. Besides, *Salmonella* possesses advantages in the preferential colonization and proliferation in tumor tissues [19], thus overcoming the major limitation associated with conventional cancer treatments. Moreover, the engineering plasticity of *Salmonella* considerably enhances its therapeutic potential through improving safety, increasing tumor targeting, and delivering anti-tumor agents [20,21]. Given these advantages, *Salmonella* has been utilized in combination with other approaches to cancer therapy, including chemotherapy, radiotherapy, and immune checkpoint inhibitors, in order to compensate for their respective deficiencies and to improve the overall therapeutic efficacy. The topic of *Salmonella* in cancer therapy has been covered in other recently published reviews [20,22,23,24,25].

In the current review, we describe the unique aspects of *Salmonella* as an anti-cancer therapeutic agent and summarize the present research on the combination of *Salmonella* with other approaches to cancer therapy, considering issues that would impact its clinical translation.

## 2. Unique Aspects of *Salmonella* in Cancer Therapy

**Figure 1:**
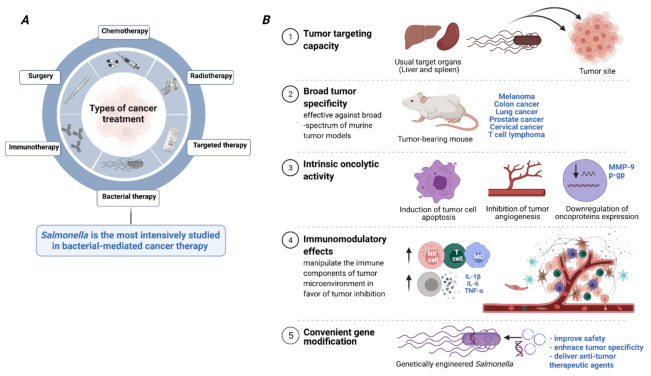
*Salmonella* characteristics that favor their use in cancer therapy. (**A**) The major modalities of cancer treatment. (**B**) The 5 key properties of *Salmonella* organisms that make them amenable for potential use in cancer therapy. The role of *Salmonella* as an anti-tumor agent has been documented against a broad-spectrum of cancers. *Salmonella*-mediated anti-tumor effects are delivered through its ability to preferentially colonize and proliferate in tumor tissues, induce direct tumor cell killing, and transform the tumor microenvironment from immunosuppressive to immunogenic. In addition, the engineering plasticity of *Salmonella* considerably enhances its efficacy in cancer therapy through improving safety, increasing specificity, and allowing the delivery of anti-tumor therapeutic agents specifically to the tumor site.

There are several properties of *Salmonella* organisms that make them suitable candidates for use in cancer therapy. These are highlighted in **Figure 1** and discussed in the following sections.

### 2.1. Selective Tumor Colonization

*Salmonella* are facultative anaerobic intracellular pathogens that exhibit strong preferential colonization in tumor tissues. In pre-clinical models, *S.* Typhimurium enrichment within tumors was >1000 fold greater than that observed in the usual target organs, including the spleen and liver [19,26]. Following administration, the bacterial colonization level in tumors was comparable to that of other tissues [21]. However, this similarity in the initial levels was disrupted within hours to days due to bacterial clearance from the circulatory system and other usual target organs, while bacterial proliferation continued within tumor tissues [21]. The mechanism(s) underlying selective tumor colonization is still not completely understood. It is thought that the hypoxic and poorly vascularized conditions within tumor tissues are more favorable to the colonization and proliferation of *Salmonella* [20]. This is consistent with *Salmonella*’s preferential colonization in necrotic regions, where essential nutrients are available and clearance by the immune system is impaired [27]. Interestingly, bacterial accumulation was not observed in other hypoxic non-tumor tissues, highlighting the contribution of other factors to *Salmonella*’s tumor selectivity [27]. Several studies revealed that chemotaxis plays an essential role in the migration of *Salmonella* to tumor tissues [28,29,30,31]. For example, aspartate, serine, and ribose/galactose chemoreceptors were successful in initiating chemotaxis of *Salmonella* organisms toward tumor cylindroids, with penetration of tissue and induction of necrosis [29]. Furthermore, it has been shown that motility is essential for bacterial accumulation in tumor tissues [32]. However, another study proposed that the accumulation of *Salmonella* in tumors is influenced by the tumor microenvironment, host reticuloendothelial system, and bacterial metabolism, and is independent of its chemotactic and mobile properties [33]. A third study attributed the initial colonization in tumor tissues to tumor necrosis factor alpha (TNF-α) induced by the intravenous administration of *Salmonella* [34]. The increased level of TNF-α induced an increase in blood influx to the tumor, thereby enhancing the colonization of *Salmonella* in tumor tissue [34].

Irrespective of the exact mechanism, the targeted distribution and proliferation of *Salmonella* in tumor tissues overcome the lack of tumor specificity and inadequate tissue penetration, which are the major limiting factors for the therapeutic efficacy of conventional cancer therapies. Moreover, the replication potential of this biological agent allows its administration at low dose and frequency, thereby minimizing toxicity and adverse side effects.

### 2.2. Broad Tumor Specificity

Several pre-clinical studies conducted in the past decades have reported the effective therapeutic index of *Salmonella*. This success was achieved at the level of tumor growth, metastasis, and host survival. The effectiveness of *Salmonella* treatment has been documented against a broad-spectrum of murine tumor models, including melanoma [35,36], colon cancer [37], lung cancer [38], prostate cancer [39], cervical cancer [40], metastatic T cell lymphoma [41], and others. In particular, *Salmonella*-mediated inhibition of metastasis was observed in different tumor models such as breast cancer [42], osteosarcoma [43], and dorsal spinal cord gliomas [44]. Moreover, the anti-tumor effects of *Salmonella* were demonstrated in metastatic patient-derived orthotopic xenograft (PDOX) murine models of osteosarcoma [45], melanoma [46], soft tissue sarcoma [47], and follicular dendritic cell sarcoma [48]. In addition to *Salmonella*’s efficacy against a wide range of tumor types, the different routes of *Salmonella* administration, including intravenous, intra-arterial, intraperitoneal, and intratumoral routes, have shown success in inhibiting tumor growth.

### 2.3. Intrinsic Oncolytic Capacity

Several studies have claimed that *Salmonella*-induced tumor inhibition is in part mediated by the intrinsic oncolytic activity of *Salmonella* itself. A variety of mechanisms underlie *Salmonella*’s intrinsic anti-tumor activity, all of which are mediated through the induction of tumor cell apoptosis. *In vitro* and *in vivo* studies utilized Annexin V detection, caspase-3 activity, and TUNEL assays to report on increased tumor cell death following infection with *Salmonella* and subsequent invasion [12,49,50]. It was suggested that nutrient deprivation and release of bacterial toxins could underlie apoptosis promotion [51,52]. In addition, the ability of *Salmonella* to induce autophagy was correlated to its intrinsic anti-tumor effect [13,53]. In one study, *Salmonella* induced autophagy in melanoma tumor cells in a dose- and time-dependent manner through downregulation of the AKT/mTOR pathway [13]. Other studies reported that *Salmonella* could delay tumor progression by inhibiting tumor angiogenesis [54,55,56]. The anti-angiogenic ability of *Salmonella* is derived from its capacity to downregulate the expression of HIF-α and vascular endothelial growth factor (VEGF) through the AKT/mTOR pathway [54]. The intrinsic oncolytic activity can be also attributed to nitrate reductase enzyme released from lysed *Salmonella* and its role in converting nitrate and nitrite into nitric oxide, which induces apoptosis in tumor cells [57,58]. Moreover, *Salmonella* was documented to downregulate the expression of certain oncoproteins in tumor cells including p-glycoprotein (p-gp) [59] and matrix metalloproteinase 9 (MMP-9) [60], inhibiting drug resistance and tumor metastasis, respectively.

### 2.4. Immunomodulatory Effects

**Figure 2:**
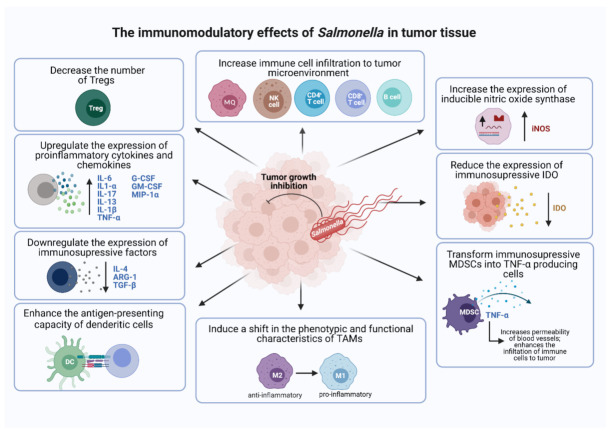
Schematic diagram illustrating *Salmonella*’s capacity to manipulate immune components of tumor in favor of tumor inhibition. Transformation of the tumor microenvironment from immunosuppressive to immunogenic occurs through increased infiltration and reprograming of anti-tumor immune cells, upregulating the expression of proinflammatory cytokines and inducing a shift in the phenotypic and functional characteristics of immune cells. The references for the summarized effects are all discussed in the text. MQ: macrophage; IL-1α: interleukin 1 alpha; IL-1β: interleukin-1 beta; G-CSF: granulocyte colony-stimulating factor; GM-CSF: granulocyte-macrophage colony-stimulating factor; MIP-1α: macrophage inflammatory protein-1 alpha; ARG-1: arginase-1; TGF- β: transforming growth factor-beta; TAMs: tumor-associated macrophages; DC: dendritic cell; M1: M1-like macrophage; M2: M2-like macrophage.

Along with the intrinsic oncolytic activity of *Salmonella*, its great ability to modulate the immune system also plays a considerable role in tumor inhibition. In this regard, it is worth mentioning that the immunosuppressive tumor microenvironment and immunosurveillance evasion mechanisms are the two major limitations in achieving effective and durable anti-cancer effects. Conventional cancer therapeutics fail to manipulate the immune component of the tumor. On the other hand, *Salmonella*-mediated cancer therapy shows remarkable success in modulating the tumor microenvironment in favor of tumor inhibition. In other words, *Salmonella* has shown its potential to shift the tumor microenvironment from immunosuppressive to immunogenic. This success is achieved through alterations in both cellular and soluble components of the immune system which, in turn, affect the phenotypic and functional properties of immune cells, as illustrated in **Figure 2**.

Several studies illustrated *Salmonella*’s ability to increase the tumor-infiltration of different innate and adaptive immune cells, including macrophages [16,61], natural killer (NK) cells [17], CD4^+^ helper T cells [16,17,61], CD8^+^ cytotoxic T cells [17,18,61], and B cells [62]. It is suggested that the recruitment of these immune cells enhances the immune response directed against tumor cells. The enhanced recruitment of neutrophils following *Salmonella* administration was also documented [17,49,58,63], but it is still not clearly understood whether neutrophil infiltration promotes or inhibits tumor growth [64]. In addition, *Salmonella* organisms attenuate tumor-induced immunosuppression and inhibit tumor growth by reducing the number of regulatory T cells (Tregs) [65,66]. *Salmonella* was also reported to upregulate the expression of pro-inflammatory cytokines and chemokines, including IL-6, IL-1α, IL-17, IL-13, G-CSF, GM-CSF, MIP-1α, and others [67]. This upregulation was attributed to *Salmonella*’s ability to activate the NF-κB pathway [68,69,70,71]. The increased expression of the immunostimulatory factors IL-1β, TNF-α, and IFN-γ as well as inducible nitric oxide synthase (iNOS) has been observed following treatment with *Salmonella* [14,36,72,73], which is accompanied by the parallel downregulation of immunosuppressive factors such as IL-4, ARG-1, and TGF-β [36,74]. It was also reported that *Salmonella* can transform the immunosuppressive myeloid-derived suppressor cells (MDSCs) into TNF-α-producing cells [75]. In turn, TNF-α enhances the infiltration of immune cells to the tumor site by increasing the permeability of tumor blood vessels [34,76]. Kaimala et al. demonstrated *Salmonella*’s ability to reduce the immunosuppressive capacity of intratumoral myeloid cells and highlighted its capacity to induce a shift in the functional characteristics of tumor-associated macrophages (TAMs) toward pro-inflammatory functions [36,61]. Consistent with these previous findings, Phan et al. (2015) illustrated *Salmonella*’s contribution to the activation of the inflammasome pathway in association with an increase in the levels of caspase-1, IPAF, and NLRP-3 [77]. Moreover, the capacity of dendritic cells to present tumor antigens was also enhanced post treatment with *Salmonella* [78]. Others suggested that *Salmonella* is able to promote T helper 1 polarization through Toll-like receptor 4 (TLR-4), and this in turn contributes to *Salmonella*-mediated tumor inhibition [16]. Grille et al. (2014) reported a marked increase in the level of intratumoral NK cell activation as assessed by CD25 expression [17]. CD25 expression correlates with the cytotoxic activity of NK cells and plays an essential role in increasing the affinity for IL-2, which in turn enhances cell proliferation and production of cytotoxic molecules [79]. Another means by which *Salmonella* induces tumor growth inhibition is through reducing the expression of indoleamine 2,3-dioxygenase (IDO) and reversing its immunosuppressive effect [80,81]. IDO plays an important role in the activation of regulatory T cells and development of immune tolerance in effector tumor-infiltrating lymphocytes [82]. Most recently, studies have investigated the ability of *Salmonella* to alter the expression of immune checkpoints on different immune cells (discussed below). In summary, the capacity of *Salmonella* to alter the intratumoral immune cell repertoire in favor of tumor regression has been amply demonstrated by different groups of investigators in preclinical models.

### 2.5. Ease of Gene Modification

Although *Salmonella* possesses immunomodulatory and intrinsic oncolytic capacities, these advantages are not sufficient for achieving optimum therapeutic outcomes. In order to enhance the overall therapeutic index of *Salmonella* treatment alone or in combination with other therapies, several strains were engineered to improve the safety and potentiate anti-tumor efficacy. *Salmonella*’s amenable gene modification helped in improving the safety of bacterial therapy through the deletion of major virulence factors or the generation of auxotrophic mutants that are incapable to replicate efficiently in an environment deficient in specific nutrients [21]. For example, deletion of msbB gene, responsible for myristoylation of an essential component of LPS, reduced *Salmonella*’s toxicity by 10,000-fold while retaining its anti-tumor efficacy in mice [83]. It is worth bearing in mind that bacterial attenuation might occur at the expense of its ability to inhibit tumor growth, because some virulence factors underlie *Salmonella*’s intrinsic anti-tumor properties [21]. The *Salmonella* A1-R strain, an attenuated leucine-arginine auxotrophic mutant, exhibited enhanced preferential colonization in tumor tissues due to the abundance of these nutrients in the tumor microenvironment but not in normal tissues [84,85,86]. Besides improving the safety and minimizing toxicity to normal organs, *Salmonella* was also engineered to enhance the antigenicity of the tumor by delivering tumor-associated or specific antigens [74]. This in turn potentiates the anti-tumor immune response in favor of tumor regression. In addition, several studies documented the unprecedented success of engineered *Salmonella* in transporting various anti-tumor therapeutic agents specifically to the tumor site. *Salmonella* has been utilized as a delivery vehicle of various cytotoxic agents (e.g., cytolysin A, PE38, and diphtheria toxin) and apoptosis-inducing proteins (e.g., Fas ligand, TRAIL, and apoptin), as well as immunomodulatory cytokines (e.g., IL-2, IL-12, IL-18, and IFN-γ) [20,56,87]. Engineering of prodrug-converting enzymes into tumor-targeting *Salmonella* was also reported. The payload enzyme released by *Salmonella* metabolizes the systemically administered pro-drug into cytotoxic agent at the tumor site only, thereby minimizing the drug-related toxic effects on normal tissues [21]. An example of prodrug-converting enzyme is cytosine deaminase (CD), which converts the non-toxic 5-flourocytosine (5-FC) into anti-tumor 5-flourouracil (5-FU). The enhanced anti-tumor efficacy of CD-expressing *Salmonella* was reported using *in vitro* and *in vivo* studies [88,89]. In this context, it is worth mentioning that the application of this approach in cancer patients could have some limitations because of the ability of 5-FU to freely diffuse across the cell membrane and exert its cytotoxic activity in neighboring cells [90]. Moreover, increased tumor targeting could be achieved by utilizing engineered *Salmonella* in order to minimize bacterial-related toxicity or enhance its therapeutic potential while keeping toxicity to minimal. For example, the surface expression of antibody fragments against specific tumor-associated antigens, including carcinoembryonic antigen (CEA) and CD20, was shown to increase *Salmonella*’s tumor localization in murine adenocarcinoma and human lymphoma models, respectively [91,92]. In addition, *Salmonella*’s engineering plasticity helped to integrate controlled gene expression systems into tumor-targeting bacteria. This could be achieved through either the incorporation of gene promoters responsive to tumor-associated signals, such as hypoxia-inducible promoter systems (HIP-1 and NirB) [93,94,95], or remotely through inducible promoters (e.g., as pBAD, pTet, and Pm) in response to exogenous transcriptional factors (e.g., l-arabinose, tetracyclines, and acetyl salicylic acid) [96,97,98,99]. This enhances tumor specificity and assures the selective expression of specific genes at the tumor site.

## 3. *Salmonella* in Combination with Other Approaches to Cancer Therapy

### 3.1. Combination with Chemotherapeutic Agents

The efficacy of chemotherapy is partly limited by systemic toxicity, lack of selectivity for cancer cells, and the chances of drug resistance development. In order to overcome these limitations, the role of *Salmonella* in enhancing the efficacy of chemotherapeutic agents against different tumor models has been investigated. Bascuas et al. reported that the intra-tumoral administration of an attenuated strain of *S. typhimurium* improved the outcomes of cyclophosphamide, doxorubicin, vincristine, and prednisone (CHOP) chemotherapy in non-Hodgkin lymphoma-bearing mice, with an overall decrease in toxicity [100]. In this model, the combination therapy successfully inhibited tumor growth and prolonged animal survival compared with either treatment alone, and was associated with an increase in the percentage of CD8^+^ tumor-infiltrating lymphocytes (TILs) as well as in the cytotoxic ability of NK cells [100]. In a study using breast cancer cells, mice treated intravenously with attenuated *S. typhimurium* plus a low dose of doxorubicin exhibited a slower tumor growth rate in comparison to those treated with single therapy [101]. It is worth noting that mice treated with the maximum tolerated dose of doxorubicin showed greater delay in tumor growth, but this was at the cost of higher toxicity [101]. It was also suggested that *Salmonella/*doxorubicin combination enhances the infiltration of CD8^+^ T cells and Tregs to the tumor site and increases the ratio of Mo-MDSCs/G-MDSCs within spleen and tumor tissues [101]. A study conducted by Lee et al. documented that the intraperitoneal administration of attenuated *Salmonella choleraesuis* improved the outcome of cisplatin therapy in hepatoma- and lung tumors-bearing mice [102]. This combination induced changes in the tumor microenvironment, including an increase in the infiltrating neutrophils, CD8^+^ T cells, and apoptotic cells [102]. Kawaguchi and his group highlighted the ability of *S. typhimurium* A1-R to enhance the anti-tumor capacity of gemcitabine among nude mice bearing pancreatic cancer PDOX [103], although gemcitabine has limited efficacy in treating pancreatic cancer. Another group succeeded in utilizing an attenuated *S. typhimurium* strain VNP20009 to improve the outcome of the standard maximum tolerated dose and lose-dose metronomic cyclophosphamide chemotherapy in B16F10 melanoma-bearing mice [104]. Chen et al. also described the improved anti-tumor effect of VNP20009 combined with triptolide in a mouse melanoma model [67]. This improvement was attributed to the enhanced capacity of combination therapy to suppress tumor angiogenesis and reduce the level of serum and tumor VEGF in comparison to either treatment alone [67,104]. In contrast to Lee et al., Chen et al. showed that combination therapy reduced tumor-infiltrating neutrophils [67]. They reported that VPN20009 alone attracted neutrophils to tumor site, but this recruitment was inhibited by the anti-inflammatory compound triptolide [67]. Interestingly, the preferential accumulation of *Salmonella* in tumor was further enhanced by the combination therapy in comparison to *Salmonella* alone [67,102,104]. This could be explained by the ability of the *Salmonella*–chemotherapy combination to reduce tumor microvascularization and provide a more hypoxic microenvironment that is conducive to *Salmonella*’s colonization and proliferation [67,104]. Moreover, a study that utilized the combination of attenuated *S. typhimurium* and carboplatinum against metastatic cancer of unknown primary (CUP) reported the ability of the combination to significantly inhibit tumor growth compared to monotherapy treatment [105]. **Table 1** shows a summary of representative studies that utilized *Salmonella* in combination with chemotherapeutic drugs to treat cancer.

Several potential mechanisms have been advanced to explain the demonstrably superior outcomes of *Salmonella* and chemotherapeutic agents in combination. Chang et al. showed that the anti-tumor effect of cisplatin was augmented when combined with *Salmonella choleraesuis* due to the capacity of *Salmonella* to increase the expression of connexin 43 (Cx43), therefore enhancing gap junction intercellular communication (GJIC) [106]. The expression of Cx43 is decreased in a variety of cancer cells and this may interfere with the response of tumor cells to treatments [107]. *Salmonella* overcomes this limitation by upregulating and activating the expression of Cx43 [78,106], thereby enhancing antigen presentation by dendritic cells [78] and facilitating the transmission of antitumor drugs and apoptosis signals between adjacent cancer cells [108]. Another study conducted by Yang et al. reported that *Salmonella choleraesuis* reduced the expression level of multi-drug resistance protein P-gp on tumor cells [59]. The decline in P-gp expression was accompanied by a decrease in its efflux capabilities and was attributed to the inhibition of p-AKT, p-p70S6K, and p-mTOR levels during *Salmonella* infection [59]. Given that high levels of P-gp on tumor cells are associated with chemotherapeutic drug resistance [109], the ability of *Salmonella* to regulate the expression of P-gp may enhance the sensitivity of tumor cells to chemotherapy. Chih’s group demonstrated the ability of *Salmonella choleraesuis* to enhance the susceptibility of B16F10 and 4T1 cells to 5-fluorouracil therapy, as illustrated by the improved therapeutic effect of the combination in tumor-bearing mice [59]. It has been suggested that *S. typhimurium* could also improve the chemosensitivity of tumor cells by inducing the quiescent cancer cells from the G_0_/G_1_ phase to the S/G_2_/M phase in the cell cycle [110,111]. In one study, almost 90% of tumor cells in the center and 80% of the total cells in an established tumor were shown to be in G_0_/G_1_ phase of the cell cycle [112]. Given that cytotoxic agents are only effective in killing proliferating cancer cells with minimal effect on quiescent cancer cells [112], *Salmonella* could enhance the chemosensitivity of quiescent tumor cells by decoying them from a chemo-resistant G_0_/G_1_ phase to a chemo-sensitive S/G_2_/M phase. This combination improved the therapeutic efficacy of cisplatinum against the osteosarcoma PDOX lung metastasis model [45]. In this study, *Salmonella* strain A1-R was utilized to enhance the sensitivity and push quiescent tumor cells to S/G_2_ phase, while recombinant methioninase was used to selectively trap the cells in this stage [45]. Finally, chemotherapy was administered to tumor-bearing mice, and this combination resulted in a remarkable tumor reduction compared to monotherapy and bitherapy [45]. In this context, the new paradigm in cancer therapy was referred to as “decoy, trap and shoot (kill)” chemotherapy [45,111].

### 3.2. Combination with Radiotherapy

Radiotherapy is a conventional cancer treatment in which ~50% of cancer patients receive radiation during the course of the disease [113]. However, radiotherapy-associated limitations obstruct achieving favorable therapeutic outcome without causing toxicity to normal tissues. *Salmonella* has been combined with radiotherapy aiming to enhance the overall efficacy of the treatment. In a study conducted in 2014, Yoon et al. utilized *S. typhimurium* to increase the radiosensitivity of the radio-resistant melanoma model [114]. They documented the success of *Salmonella* and gamma radiation combination and its ability to induce greater apoptosis in B16F10 cells in comparison to radiotherapy alone or *Salmonella* alone [114]. They also showed that the combinatorial treatment resulted in a significant inhibition of tumor growth in melanoma-bearing mice, and a prolonged survival rate compared to monotherapy [114]. Other groups performed studies in which engineered *Salmonella* was combined with radiotherapy. The combination of lipid A mutant *S. typhimurium* and X-rays resulted in a supra-additive therapeutic effect in B16F10 or Cloudman S91 melanoma-bearing mice [115]. Other studies demonstrated an improved CT26 tumor growth inhibition when treated with a combination of *S. typhimurium* strain ΔppGpp/pBAD-ClyA and radiotherapy, highlighting the contribution of radiotherapy to the colonization of *Salmonella* in tumor tissues [116]. Chen et al. intended to improve specificity and anti-tumor therapeutic ability against malignant melanoma by taking advantage of the preferential colonization of *Salmonella* strain VNP20009 in the hypoxic tumor tissues and coating this bio-therapeutic agent with the photothermal agent polydopamine [117]. In this therapy, the systemic administration of *Salmonella* helped in the delivery of polydopamine to hypoxic and poorly vascularized regions of the tumor. Thereafter, when tumor tissue was irradiated, polydopamine helped in the conversion of near-infrared light into heat, which then increased the local temperature and eventually killed the surrounding cancerous cells, leading to tumor growth inhibition. The authors brought attention to the ability of this combination to substantially eliminate melanoma tumors without relapse [117]. The combinatorial therapy also resulted in a higher level of apoptosis in melanoma tumors and a greater induction of cytokine secretion, which contributed to the enhanced anti-tumor effect. Attenuated *Salmonella typhi* Ty21a was also an excellent vehicle to deliver gold nanoparticles to the radio-resistant central hypoxic regions of CT-26 colon cancer [118]. Gold nanoparticles have been shown to enhance the efficacy of radiotherapy [119]. Thereby, when these particles reach hypoxic regions, this compensates the low anti-tumor efficacy of radiotherapy at tumor hypoxic sites. From an immunological perspective, *Salmonella*-induced changes in the immune component of tumor microenvironment could underlie its ability to enhance tumor radiosensitivity. Barker et al. proposed various immunological mechanisms for radiosensitization, including: (a) increasing the number and activation levels of dendritic cells, (b) effective T-cell recruitment and activation, (c) inhibition of T-cell exhaustion and abrogation of inhibitory signaling, and (d) induction of specific cytokine (GM-CSF, IL-2 and IL-12) and chemokine (CCL3 and CCL5) release [120]. We have previously discussed the role of *Salmonella* in manipulating both innate and adaptive components of the immune system and highlighted its potential in immunomodulating the tumor niche. The capacity of *Salmonella* to transform the tumor microenvironment from being immunosuppressive to be immunogenic makes it a good candidate for improving the sensitivity of tumor to radiotherapy.

### 3.3. Combination with Immune Checkpoint Inhibitors

There is no doubt that the development of immune checkpoint inhibitors is considered a paramount achievement in cancer immunotherapy. Checkpoint inhibitors mainly work by targeting the negative regulators of T cell function, such as CTLA-4, PD-1, and PD-L1, unleashing T-cells and allowing them to induce tumor cell death. The past few years have witnessed unprecedented and promising therapeutic outcomes in different types of cancer, including advanced melanoma [121,122] and non-small cell lung carcinoma [123,124]. Despite the outstanding success reported with immune checkpoint inhibitors, the percentage of cancer patients who respond to this approach remains rather modest [125], and serious immune-related adverse events were observed in some patients receiving checkpoint inhibitors therapy [126,127]. This observation has led to the identification of different predictive biomarkers that help in patient selection, such as (a) the presence of tumor-infiltrating lymphocytes [128,129,130,131], (b) the expression level of checkpoint inhibitors [132,133], and (c) the tumor mutational load [134,135].

Different studies have evaluated the effect of *Salmonella* on the expression of inhibitory checkpoint proteins in different tumor and non-tumor cells. Chen et al. reported that *Salmonella choleraesuis* dose-dependently downregulated the expression of PD-L1 on different murine and human cancer cell lines through inhibiting the AKT/mTOR/p70s6K signaling pathway [136]. They also showed that the systemic administration of *Salmonella* decreased the expression of PD-L1 in B16F10 and LL2 tumor tissues [136]. This downregulation enhanced T cell infiltration and thereby resulted in tumor growth inhibition [136]. Another study documented the role of attenuated shIDO-ST (*S. typhimurium* delivering an shRNA plasmid targeting IDO) in inhibiting the expression of several inhibitory checkpoint proteins, including PD-L1, PD-1, and CTLA4, in different splenic immune cells [137] which highlights the capacity of *Salmonella* to restructure the immune component of the tumor microenvironment in favor of tumor inhibition. On the other hand, different studies reported *Salmonella*-mediated upregulation of PD-L1 in different cell types. Newland et al. utilized transcriptome analysis and flowcytometry to study the effect of *S. typhimurium* on PD-L1 expression in NOD mice [138]. In their model, the intravenous administration of *Salmonella* significantly increased the proportion and surface expression of PD-L1 among dendritic cells and macrophages [138]. Others also reported an increase in PD-L1 expression in B cells and CD4^+^T cells following *Salmonella* treatment [139,140,141]. It is worth mentioning that none of these studies correlated the increased PD-L1 expression with tumor growth inhibition.

Binder et al. elegantly demonstrated the capacity of ovalbumin-producing *S. typhimurium* A1-R and anti PD-L1 combination to rescue the function of peripheral and tumor infiltrating CD8^+^ T cells against B16-OVA melanoma [142]. This led to an increase in tumor rejection compared to bacterial therapy alone or the combination of anti PD-L1 and anti-CTLA-4 [142]. The same study reported the failure of *Salmonella* and anti CTLA-4 combination in treating tumor-bearing mice. Zhao et al. combined bacterial therapy with immune checkpoint inhibitors by transforming attenuated *S. typhimurium* with a plasmid for RNA interference targeting the inhibitory receptor PD-1 (siRNA-PD-1), and used it in B16 melanoma [143] and CT26 colon cancer [144] models. They reported a preferential accumulation of transformed *Salmonella* in tumor tissues [143] and found that the intratumoral injection of siRNA-PD-1-carrying *Salmonella* resulted in (a) a remarkable decrease in tumor weight, (b) prolonged survival rate of tumor bearing mice, in addition to (c) an increase in CD4^+^ and CD8^+^ TILs compared to treatment with *Salmonella* containing a plasmid for scrambled si-RNA [143,144]. It is worth noting that a comparison with anti-PD-1 alone was not performed. In another study of colon cancer, CT26 or MC38 tumor-bearing mice treated with a combination of *Salmonella* carrying IDO siRNA and anti-PD-1 showed delayed tumor growth in comparison to anti PD-1 monotherapy [145]. However, the combination showed no additional tumor growth inhibition compared to *Salmonella* treatment alone [145]. A recent study also used sub-therapeutic doses of IDO-targeting *S. typhimurium* along with anti-PD-1/CTLA-4 antibodies to treat LLC1-tumor bearing mice [137]. They observed a significant delay in tumor growth in mice that received the treatment combination in comparison to those received either treatment alone, and this inhibition was associated with an increase in tumor infiltration of CD4^+^ and CD8^+^ T cells [137]. These results collectively highlighted *Salmonella*’s capacity to enhance the efficacy of checkpoint inhibitors in treating tumor-bearing mice. However, the immunological response underlying the enhanced outcome of *Salmonella*-checkpoint inhibitors combination did not receive sufficient attention and need to be further investigated.

### 3.4. Combination with Immunomodulatory Cytokines

The approval of using immunomodulatory cytokines as a part of cancer therapeutics dates back to late 20th century, when IL-2 was approved by the USFDA for treatment of metastatic kidney cancer and metastatic melanoma [146] and IFN-alpha for hairy cell leukemia [147]. In Europe, TNF was licensed for treating irresectable soft tissue sarcoma. Nowadays, these cytokines are no longer used in clinical applications due to the severe toxicities associated with their systemic administration and their limited effectiveness [3,148]. *Salmonella*’s ability to specifically target tumor tissues and the fact that it can be easily genetically modified raised the notion of utilizing *Salmonella* as a vector to carry immunomodulatory agents to tumor tissues and express them under a controlled expression system. For example, the oral administration of *S. typhimurium* expressing human IL-2 was successful in enhancing the anti-tumor effects and reducing the number of metastasis in mice with unresectable hepatic malignancies [87]. It was also shown that CD8^+^ T cells and NK cells were responsible for the antitumor activity [149]. This was also shown in other studies that utilized *Salmonella* carrying IL-2 gene for the treatment of murine osteosarcoma [150] and melanoma [56]. In a canine osteosarcoma model, *Salmonella* expressing IL-2 resulted in a prolonged disease-free interval [151]. In patients with metastatic gastrointestinal cancer, attenuated *Salmonella* carrying IL-2 resulted in an increase in circulating NK and NKT cells but with no survival advantage [152]. In the B16F10 melanoma model, subcutaneous administration of *S. typhimurium* expressing IFN-γ resulted in efficient tumor inhibition and prolonged survival mainly driven by the NK cell response [153]. *S. typhimurium* harboring TNF-α also showed potential against murine melanoma, with an ability to enhance the outcome of other cancer therapeutics [154]. In a study conducted by Loeffler et al., *Salmonella* expressing the chemokine CCL21 significantly inhibited the growth of multi-drug resistant murine carcinomas in a CD4^-^ and CD8^-^ cell-dependent manner. This inhibition was associated with elevated levels of IFN- γ, CXCL9 and CXCL10 [155]. Several other studies [156,157,158,159] utilized different cytokine-expressing bacteria and emphasized the remarkable potential of *Salmonella* to deliver immunomodulators in an efficient and well-tolerated manner.

### 3.5. Combination with Other Therapies

Along with the previously mentioned promising approaches, *Salmonella* could be also combined with other therapies, including adoptive T cell transfer [160], anti-angiogenesis therapy [161], caffeine and valproic acid [162], and a traditional Chinese medicine herbal mixture [163], to improve anti-tumor effects. An interesting study demonstrated that T cell transfer in combination with either the intravenous administration of live bacteria or intratumoral treatment with heat-killed bacteria resulted in long-established tumor eradication and relapse prevention, thereby improving the outcome of adoptive T cell transfer [160]. It is worth noting that this enhanced outcome was driven by the increased number of neutrophils and decreased number of monocytes in the tumor microenvironment [160], which have been thought to enhance T cell therapeutic efficacy [164]. In another study, significant tumor growth inhibition was observed when *S. typhimurium* A1-R was utilized in combination with caffeine and valproic acid against pleomorphic rhabdomyosarcoma PDOX model, and this inhibition was greater than that observed with cyclophosphamide treatment [162]. Moreover, the combination of *S. typhimurium* and traditional Chinese medicine herbal mixture also showed potential in treating aggressive types of cancer compared to monotherapies [163].

## 4. Clinical Application of *Salmonella* in Combination Cancer Therapy

### 4.1. Clinical Trials

Several genera of bacteria have been studied in pre-clinical settings, and a few bacterial platforms have been selected for testing in human patients, namely *Listeria monocytogenes, Clostridium novyi*, and *S. typhimurium*. These bacterial species have received the most attention due to the increased understanding of their physiology, pathogenicity, and genetics which, in turn, led to the development of attenuation strategies critical for safe administration of live bacteria in humans. Among bacterial species tested in clinical settings, strains of *Listeria monocytogenes* have shown encouraging and promising results [21]. Besides, clinical signs of tumor colonization and objective evidence of tumor responses have been observed following the intravenous and intratumoral administration of *Clostridium novyi*-NT spores [165,166,167]. The observed tumor destruction and absence of viable tumor cells were attributed to gas pockets produced by *Clostridium novyi*-NT. However, treatment with oncolytic bacteria alone failed to eradicate all cancer cells, and thereby led to tumor progression or recurrence [166,168].

The first *Salmonella* strain studied in human clinical trials was *S. typhimurium* VNP20009, and was tested against metastatic melanoma and metastatic renal carcinoma. The study revealed that the maximum tolerated dose was 3.0 × 10^8^ CFU/m^2^ injected through the intravenous route. Despite the observed tumor colonization and increased pro-inflammatory cytokines in some of the treated patients, no objective tumor regression was reported [169]. Another clinical trial utilized *S. typhimurium* VNP20009 to treat metastatic melanoma patients. Consistent with previous findings, no tumor regression was documented among treated patients [170]. In order to improve therapeutic efficacy, VNP20009 was engineered to express the *E coli* CD enzyme, which converts the non-toxic 5-FC into anti-tumor 5-FU, and was tested in three patients with head and neck squamous carcinoma and esophageal adenocarcinoma through intratumoral injection. Tumor colonization was observed in two patients for at least 15 days post administration, with a 3:1 tumor-to-plasma ratio of 5-FU; this ratio was <1.0 in the non-colonized patient. No adverse effects were reported following the treatment [171]. Later trials utilized the oral route for *Salmonella* administration. *S. typhimurium* Ty21a that expresses VEGFR2 (VXM01 vaccine) has been used against advanced pancreatic cancer. Although the engineered bacteria succeeded in enhancing VEGFR2-specific T cell effector responses and reducing tumor perfusion, adverse events such as neutrophilia, lymphopenia, and diarrhea were observed in treated subjects [172]. SalpIL2 (*S. typhimurium* that expresses human IL-2) showed no significant benefits in phase I clinical study against metastatic gastrointestinal cancer. On the other hand, an increase in circulating NK cells and NK-T cells was reported among treated patients [152], which provides insights into the possibility of combining *Salmonella* strains with other approaches of cancer immunotherapy. **Table 2** summarizes previous and ongoing clinical trials using *Salmonella*-based cancer therapy. The results of clinical trials highlighted the discrepancy in the therapeutic outcome between pre-clinical and clinical models. This could be related to differences in tumor contextures and growth rates that might alter bacterial invasiveness and proliferation within tumor tissues [173]. Others attributed the inefficiency of BMCT in clinical trials to the over-attenuation of the utilized bacteria which, in turn, compromises its anti-tumor effects [174].

### 4.2. Challenges

Despite the success reported with the use of *Salmonella* as an integral part of combination therapies, the translation of this success from pre-clinical to human clinical application still poses major challenges. The limitations associated with the clinical application of combinatorial therapies mirror those related to the implementation of *Salmonella* therapy alone. Although BMCT dates back to the mid-19th century, the introduction of *Salmonella* to human cancer treatment remains pending due to several challenges, including:

- **Clinical safety.** Bacterial pathogens can cause diseases through the action of virulence factors. On the other hand, the attenuation of virulence factors has been correlated with decreased anti-tumor therapeutic effects [183,184]. The optimized balance between reduced virulence and clinical efficacy remains the major challenge in the clinical application of BMCT. Moreover, not all bacterial strains that showed success in pre-clinical models can be used in clinical settings due to their distinct pathologies that differ in animals and humans.
- **Route of administration.** The systemic administration of bacteria increases the risk of toxicity and potential adverse effects of the infection. The oral administration is considered relatively safer but at the expense of therapeutic efficacy.
- **Dose optimization.** Since live bacteria proliferate in target tissues, the effective dose does not necessarily mirror the administered dose. The effective dose is dependent on different factors including the route of administration, accessibility to target tissues, level of vascularization, tumor immunogenicity, and the presence of tumor-infiltrating inflammatory cells [21].
- **Genetic instability.** Live genetically engineered bacteria that carry antibiotic resistance genes or mobile genetic elements are not suitable for clinical use since these recombinant elements can mediate horizontal gene transfer [185]. Other recombinant plasmids can be lost or mutated before reaching tumor tissues, leading to exaggerated infection or therapeutic failure [186].
- **Bacterial growth control *in vivo*.** The timely elimination of bacteria using an antibiotic intervention is critical since the early administration of antibiotic may eliminate the infection before an anti-tumor effect has been achieved, whereas a late intervention would result in unpredictable systemic inflammatory response. Microbiota disturbance and the development of antibiotic resistance should be kept in mind when using antibiotics to manage bacterial growth.
- **Tumor recurrence.** Despite the considerable role of bacteria in treating cancer, subsequent tumor recurrence could still be possible [14]. This may be attributed to the activation of immune tolerance or evasion mechanisms that interfere with the bacteria-mediated immune response [25].
- **Patient selection.** Chemotherapeutic agents suppress the immune system and interfere with delivering the immunomodulatory effects induced by BMCT. Therefore, the risk of bacterial infection is substantially increased in chemotherapy-receiving patients. In addition, brain abscesses, diverticulitis, or recent radiation might enhance the unintentional growth of bacteria in non-target tissues. Live bacteria also have the potential to colonize the foreign bodies in patients with joint replacement, artificial heart valves, and impanated medical devices [22]. Pre-exposure and anti-bacterial immunity should be also taken into account since they might interfere with the anti-tumor immune-stimulatory effects of the bacteria and result in treatment failure.

### 4.3. Efforts to Overcome Challenges

*Salmonella*’s anti-cancer and immunomodulatory characteristics, along with its durable capacity to enhance the outcome of other modalities of cancer therapy, necessitate the development of strategies to overcome the limitations and facilitate the implementation of *Salmonella* in clinical use. In the past decades, several strategies have been followed to maintain the balance of therapeutic efficacy and safety when utilizing *Salmonella* in cancer therapy. The aim of the earliest approaches was to enhance tumor targeting by selection, and this was done by passaging *Salmonella* through cancer cells either *in vitro* or *in vivo*. The attenuated *Salmonella* strains VNP20009 and A1-R were generated following such strategy of random selection [187,188,189]. The ineffectiveness of VNP20009 in treating cancer patients was due to the over-attenuation through uncontrolled introduction of multiple deletions during the process of selection [169,174]. *Salmonella* strain A1-R showed promise in treating different types of cancer in pre-clinical models but has not been utilized in clinical trials so far [187,190]. Unexpected modifications were then avoided by designing *Salmonella* strains through targeted gene deletions. Frahm et al. and his group investigated the role of LPS in establishing the balance between the therapeutic and harmful effects of *S. typhimurium*. They concluded that minor modifications of LPS (e.g., Δ*rfaP*, Δ*rfaL*) alone did not alter the toxicity of *Salmonella*, whereas the core deletion mutants Δ*rfaG* and Δ*rfaD* considerably enhanced their safety at the expense of the anti-tumor activity [184]. To this point, they succeeded in establishing a balance between therapeutic potency and attenuation through controlling the synthesis of LPS using the inducible arabinose promoter P_BAD_ [8,184]. Targeted gene engineering was also implemented to induce modifications in Lipid A and flagella synthesis, resulting in the auxotrophic *Salmonella* vector strain SF200 (Δ*lpxR9* Δ*pagL7* Δ*pagP8* Δ*aroA* Δ*ydiV* Δ*fliF)* [191]. This attenuated strain showed enhanced immune-stimulatory capacity and overcame the limitation of pre-exposure of anti-*Salmonella* immunity. The careful selection of genetic manipulations is critical for maintaining the balance between therapeutic potential and pathogenicity. Moreover, another study suggested an approach to override the non-specific invasiveness and intrinsic toxicity of *Salmonella* through coupling the bacteria with a surface-expressed single-domain antibody directed against a tumor-associated antigen [92]. This approach showed success when CD20-targeted *Salmonella* was employed against lymphomas with minimal non-specific invasiveness [92]. Other researchers utilized *Salmonella* as a vector system for drug delivery in order to enhance its therapeutic potential and overcome the limitation in tumors that could not be resolved by the intrinsic and immunostimulatory effects of *Salmonella* alone. Interestingly, Din et al. illustrated the potential of utilizing *Salmonella* as a targeted delivery system for therapeutic agents. In their model, bacterial growth was controlled through engineering a bacterium that undergoes lysis at a threshold population density, allowing the repetitive release of genetically encoded anti-tumor therapeutic agents during lytic cycles [192]. This helps in delivering the therapeutic benefit while minimizing the systemic inflammatory response [192]. In this context, it would be ideal if bacterial presence could be controlled, allowing sufficient time to achieve the optimal anti-tumor effects and then eliminating it to avoid uncontrolled proliferation that may lead to unpredictable systemic inflammatory responses. Another innovative step in designing therapeutic vectors was taken by Mercado-Lubo et al. through conjugating gold nanoparticles (AuNPs) with the effector protein SipA of *Salmonella* SPI1 (*Salmonella* pathogenicity island 1) [193]. SipA protein induces caspase-3-mediated cleavage of the multi-drug resistance p-gp and inhibits its function. Despite the observation that the SipA-AuNPs nanoparticle did not result in any therapeutic effects when tested in murine tumor models of colon and breast cancers, the enhanced anti-tumor efficacy was observed when combined with doxorubicin. The therapeutic benefit was delivered by the capacity of the SipA protein to abrogate the function of p-gp and therefore retrieve the desired effect of doxorubicin. The concept of applying nanoparticles to deliver *Salmonella* proteins is well-accepted in circumventing the safety concern of using infectious agent like *Salmonella*, but nanoparticles lack the unique feature of *Salmonella* in the preferential colonization of tumor tissues. A recently published paper utilized nanoparticle technology and *Salmonella* to enhance the treatment efficacy and safety [194]. They employed silver nanoparticles (AgNPs) conjugated with sialic acid to locally deplete tumor-infiltrating neutrophils through the selective recognition of L-selectin, thereby enhancing the efficacy of *Salmonella*. AgNPs also exert their function through inducing direct tumor cell killing and clearing *Salmonella* following tumor eradication to minimize the undesirable side effects. This way, the combination of *Salmonella*-AgNPs resulted in a superior therapeutic outcome. Furthermore, controling gene expression, either remotely or through the incorporation of gene promoters responsive to tumor-associated antigens, has shown a good potential in minimizing toxicity to normal tissues (discussed in Section 2.5).

In combinatorial therapies that involve bacterial treatment, high doses of bacteria are not necessarily correlated with a better outcome. Bacteria can be utilized at a low dose that is capable to induce changes in the immune components of the tumor microenvironment without any significant tumor inhibition effect. This way, it may enhance the therapeutic outcome and increase the efficacy of other treatments while keeping toxicity to minimal. As previously mentioned, the therapeutic index of bacterial therapy in pre-clinical model has been enhanced over the past few years with the designation of engineered bacteria. While retaining tumor specificity and anti-tumor efficacy, bacteria have been engineered for the purpose of (a) improving safety, (b) enhancing tumor targeting and minimizing toxicity to normal tissues, and (c) delivering various anti-tumor therapeutic agents such as cytokines, cytotoxic agents, tumor-associated antigens, and prodrug enzymes [21]. Taken all together, the durable success achieved with combination therapies (as compared to monotherapies) in pre-clinical models has increased the demand to overcome the challenges associated with bacterial therapy. This, in turn, will pave the way for implementing combination therapies involving *Salmonella* in clinical use.

## 5. Conclusions

**Figure 3:**
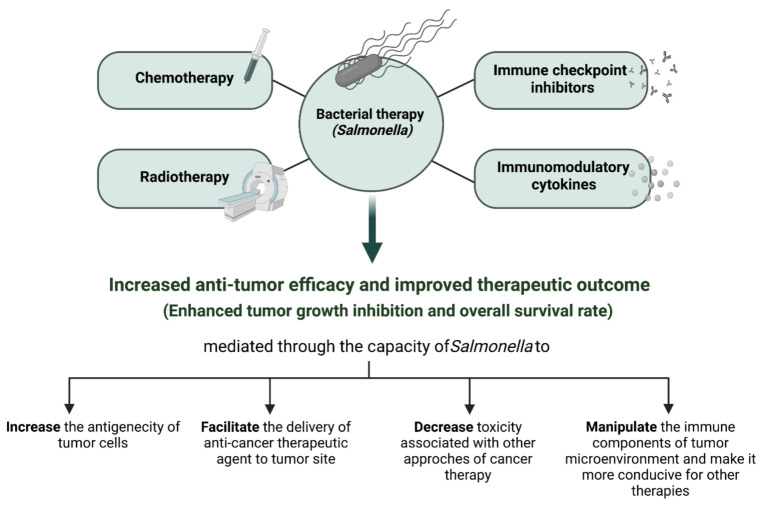
Combination treatments involving *Salmonella* in cancer. The administration of *Salmonella* was successful in improving therapeutic outcome of other conventional and non-conventional cancer treatments.

The present review highlights the unique characteristics of *Salmonella* as a potential anti-cancer therapeutic agent. The essential tumor targeting capacity, adequate tumor tissue penetration, immunomodulatory effects, and extensive gene packaging capacity, in addition to efficacy in delivering anti-tumor therapeutic agents, suggest *Salmonella* as a good candidate for combination therapy in cancer. Several pre-clinical studies have demonstrated the enhanced anti-tumor efficacy of conventional and non-conventional cancer therapeutics in combination with attenuated *Salmonella* in different tumor models. The improved therapeutic outcome was shown in terms of tumor growth retardation, metastasis inhibition, and increased survival rate. Taking everything into consideration, the synergistic effect of combinatorial therapy is thought to be achieved through the ability of *Salmonella* to (a) improve the delivery and enhance specificity of cancer therapeutic agents to tumor site, (b) increase the antigenicity of tumor cells, and (c) manipulate the immune components of the tumor microenvironment to make it more conducive for improving other therapies, along with its ability to (d) minimize the toxicity associated with other cancer therapies either directly or indirectly by reducing the number of treatment cycles or prolonging the effect of a single treatment (**Figure 3**).

Although the potential of utilizing *Salmonella* to enhance the outcome of other therapies has been investigated, the mechanistic details underlying combination therapies are still scarce. Additional research is needed to expand the current findings and study the underlying mechanism(s) by which *Salmonella* improves the therapeutic outcome. Moreover, the modulation of the immune system in combination cancer therapy is worthy of further study because of its essential involvement in cancer development and progression. The increased understanding of the involvement of *Salmonella* in combinatorial therapies will facilitate the clinical translation of such an approach, thereby potentially ushering in a breakthrough in cancer therapy.

## Figures and Tables

**Table 1:** Representative pre-clinical examples of *Salmonella*-chemotherapy combination in treating cancer.

Chemotherapeutic Agent	*Salmonella* Strain	Route of *Salmonella* Administration	Cancer Model/ Mouse Strain	Outcome of Combination Treatment	Associated Mechanism	Ref
CHOP chemotherapy (a cyclophosphamide, doxorubicin, vincristine, and prednisone/steroid combination)	*S. typhimurium* LVR01	Intratumoral	B-cell non-Hodgkin lymphoma (murine A20 cell line)/BALB/c mice	Delayed tumor growth compared to monotherapy alone Prolonged overall survival and progression-free survival of tumor-bearing mice Improved overall health status of mice undergoing chemotherapy	Increase in the % of intratumoral CD8^+^ T cells Increase in the infiltration of neutrophils and NK cells in tumors (compared to untreated and chemotherapy-receiving mice) Enhanced NK cell-mediated cytotoxicity Improved anti-A20 specific antibody immune responses (compared to untreated and chemotherapy-receiving mice) Upregulation in Cxcl1 gene expression	[100]
Doxorubicin	*S. typhimurium* DSLpNG	Intravenous	Autochthonous model of breast cancer/BALB/neuT mice	Inhibited tumor growth rate more efficiently than *Salmonella* alone or low-dose doxorubicin alone No clinically relevant toxicity was reported in treated mice	Increase in the infiltration of CD8^+^ T cells and Treg cells in tumors Increased Mo-MDSCs/G-MDSCs ratio within spleen and tumor tissues (this was observed in *Salmonella*-treated and combination-treated groups, but not among doxorubicin-receiving mice)	[101]
Cisplatin	*S. choleraesuis*	Intraperitoneal	Lung tumor (murine LL/2 cell line)/C57BL/6 mice and hepatoma (murine ML-1 cell line)/BALB/c mice	Additively retarded tumor growth in both highly aggressive lung tumor and slowly growing hepatoma models Prolonged survival time	Combination therapy promoted the preferential accumulation of *Salmonella* within lung tumors An increase in the number of infiltrating CD8^+^ T cells, neutrophils, and apoptotic cells was observed in tumor tissues	[102]
Gemcitabine	*S. typhimurium* A1-R	Intravenous	Pancreatic cancer PDOX mouse model/nude mice	Inhibited tumor growth compared to monotherapy No effect on mice body weights was observed	Extensive necrosis was observed in tumor tissues from gemcitabine–*Salmonella* receiving mice in comparison to untreated mice	[103]
Cyclophosphamide	*S. typhimurium* VNP20009	Intraperitoneal	Melanoma model (murine B16F10 cell line)/C57BL/6 mice	Remarkable tumor growth inhibition was observed when *Salmonella* was combined with MTD and LDM chemotherapy Prolonged overall survival	Decrease in tumor microvessel density Increase in serum levels of VEGF Increase in the number of bacteria within tumors when compared with bacterial treatment alone	[104]
Triptolide	*S. typhimurium* VNP20009	Intraperitoneal or intratumoral	Melanoma model (murine B16F10 cell line)/C57BL/6 mice	Suppressed melanoma tumor growth (through both intraperitoneal and intratumoral routes of bacterial administration) Remarkably prolonged the survival length of tumor-bearing mice (through the intraperitoneal injection of *Salmonella*)	Enhanced tumor localization of *Salmonella* Increase in the number of necrotic tumor cells Decrease in the infiltration of inflammatory cells to tumors Angiogenesis inhibition (reduction in the number of CD31^+^ cells and inhibition of VEGF expression)	[67]
Carboplati-num	*S. typhimurium* A1-R	Intravenous	Cancer of unknown primary PDOX model/ nude mice	Suppressed tumor growth compared to each treatment alone	Reduced tumor cell size and cellularity Extensive cytoplasmic vacuolization in tumor cells was observed	[105]

(Mo-MDSCs: monocytic myeloid-derived suppressor cells; G-MDSCs: granulocytic myeloid-derived suppressor cells; MTD: maximum tolerated dose; LDM: low-dose metronomic).

**Table 2:** Summary of previous and ongoing bacterial-therapy clinical trials utilizing *Salmonella*.

*Salmonella* Strain	Route of Administration	Cancer Model	Phase	Recruitment Status	Identifier (Nct Number)	Ref
VNP20009	Intratumoral	Refractory, superficial solid tumors	I	Completed	NCT00004216	[175]
VNP20009	Intravenous	Advanced or metastatic cancer	I	Completed	NCT00004988	[176]
VNP20009	Intravenous	Metastatic melanoma and metastatic renal cell carcinoma (RCC)	I	Completed	NCT00006254	[177]
*S. typhimurium* SalpIL2 (expresses human IL-2)	Oral	Liver metastasis of solid tumors	I	Completed	NCT01099631	[178]
VXM01 vaccine (*S. typhimurium* Ty21a expresses VEGFR2)	Oral	Advanced pancreatic cancer	I	Completed	NCT01486329	[179]
*Salmonella* CVD908ssb strain (TXSVN vaccine)	Oral	Multiple myeloma	I	Not yet recruiting	NCT03762291	[180]
*S. typhimurium* strain (SS2017) expressing tumor DNA vaccine	Oral	Neuroblastoma	Early phase I	Recruiting	NCT04049864	[181]
Saltikva (*S. typhimurium* expresses human IL-2)	Oral	Metastatic pancreatic cancer	II	Recruiting	NCT04589234	[182]

## Abbreviations

CAR-T	Chimeric antigen receptor T cell therapies
BMCT	Bacterial-mediated cancer therapy
TNF-α	Tumor necrosis factor-alpha
PDOX	Patient-derived orthotopic xenograft
VEGF	Vascular endothelial growth factor
p-gp	P-glycoprotein
MMP-9	Matrix metalloproteinase 9
Tregs	Regulatory T cells
iNOS	Inducible nitric oxide synthase
MDSCs	Myeloid-derived suppressor cells
IDO	Indoleamine 2,3-dioxygenase
NK	Natural killer
Cx43	Connexin 43
TILs	Tumor-infiltrating lymphocytes
